# Management of patients with rare diseases in the Middle East: challenges & opportunities – insights from the Rare Advocacy Council

**DOI:** 10.1186/s13023-026-04469-1

**Published:** 2026-07-31

**Authors:** Agnès Farrugia, Ahmed Bahey, Ahmad Tarawah, Arwa Al Yamani, Carla Abou Selwan, Dania Mohty, Denis Wolfs, Dorica Dan, Hafiz Mosa Ali Malhan, Johan De Graaf, Stefan Živković, Yasser Wali, Youmna Ouraybi, Zakareya Al Kadhem, Nafisa Tawfiq, Lee Davelaar, Noha Mohamed Mahmoud Abdelbaky

**Affiliations:** 1 Association Française Contre L’Amylose, Marseille, France; 2 Qatar Friends Hemophilia Group (QFHG), Doha, Qatar; 3 Pediatric Hematology-Oncology, King Salman Medical City, Madinah, Saudi Arabia; 4 Al Madinah Hereditary Blood Disorders Charity Society, Madinah, Saudi Arabia; 5 MENA Hematology League, Dubai, United Arab Emirates; 6 Hematology/Oncology/BMT Deputy Chairman of Saudi and Thalassemia and Sickle Cell Anemia Society, Riyadh, Saudi Arabia; 7 Saudi Association of Pediatric Hematology/Oncology Society (SAPHOS), Riyadh, Saudi Arabia; 8 Science PRO, Jal-el-dib, Lebanon; 9 King Faisal Specialist Hospital, Riyadh, Saudi Arabia; 10 Amyloidosis Association, Brussels, Belgium; 11 Romanian National Alliance for Rare Disease, Bucharest, Romania; 12 Adult Hematologist, Riyadh, Saudi Arabia; 13 Dutch Pituitary Foundation, Nijkerk, The Netherlands; 14 Endo-ERN European Reference Network on Rare Endocrine Conditions, Leiden, The Netherlands; 15 Department of Endocrinology, Leiden University Medical Center, Leiden, The Netherlands; 16 National Organization of Rare Diseases, Belgrade, Serbia; 17 Pediatric Hematology, College of Medicine and Health Sciences, Sultan Qaboos University, Board Member Omani Society of Hematology, Muscat, Oman; 18 Ana Fareed Health Consultancy for Patient Advocacy, Dubai, United Arab Emirates; 19 Bahrain Society for Sickle Cell Disease Patients Care, Manama, Bahrain; 20 UAE Rare Disease Society, Dubai, United Arab Emirates; 21 Emerging Markets, Global Policy & Public Affairs, Rare Diseases, Pfizer, Sydney, Australia; 22 Regional Patient Advocacy – Middle East, Pfizer, Cairo, Egypt

**Keywords:** Diagnosis and treatment access, Healthcare policy, Middle East, Multidisciplinary care, Patient advocacy, Rare diseases

## Abstract

**Background:**

Rare diseases affect a small percentage of the population but collectively impact millions worldwide. In the Middle East, the challenges are intensified by regional factors such as high rates of consanguinity, sociocultural stigma, limited diagnostic capacity, and inadequate healthcare infrastructure. These challenges often lead to delayed diagnoses, restricted access to treatment, and poor quality of life for affected individuals and their families.

**Methods:**

The Rare Advocacy Council conducted two 1.5-hour virtual expert panels involving 14 regional and international stakeholders (5 clinicians, 4 patient advocates, and 5 international academic experts) to identify and prioritize the challenges of managing rare diseases in the Middle East region. Discussions were organized across four domains: disease recognition and diagnosis, the patient journey and continuum of care, access to timely diagnostics, and access to adequate treatment, followed by structured online voting (involving only clinicians and patient advocates; *n* = 9), discussions focused on prioritization, and a descriptive follow-up survey to identify the most critical barriers and propose actionable solutions.

**Results:**

Key challenges identified included the lack of national disease registries, limited public awareness, underrepresentation of patient voices in decision-making, fragmented multidisciplinary care, and restricted access to diagnostics and advanced therapies. Top priorities included developing national registries, enhancing media-driven education, strengthening collaboration among care providers, and improving treatment accessibility through policy reforms.

**Conclusions:**

Effective management of rare diseases in the Middle East requires a coordinated, patient-centered approach. Strengthening health system infrastructure, investing in education, and aligning policy with patient needs are essential for sustainable improvement. Collaborative action among policymakers, healthcare providers, and advocacy groups can significantly advance care delivery and improve outcomes for individuals living with rare diseases.

**Supplementary Information:**

The online version contains supplementary material available at 10.1186/s13023-026-04469-1.

## Introduction

Rare diseases, often referred to as orphan diseases, represent a diverse group of conditions that affect a relatively small portion of the population but collectively impact millions worldwide [1, 2]. These conditions are frequently chronic, progressive, and life-threatening, posing significant challenges in diagnosis, treatment, and long-term management [1–3]. Rare diseases are typically defined as conditions with a very low prevalence, affecting fewer than 1 in 2,000 to 10,000 individuals [4]. This limited prevalence has significant implications for research funding, drug development, and healthcare policy.

Despite efforts to raise awareness and promote advocacy for rare disease research, the cumulative burden of rare diseases remains substantial [5–7]. Many patients experience lengthy diagnostic delays, misdiagnoses, and restricted access to effective treatments due to gaps in robust epidemiological data and limited investment in orphan drug development [2, 3]. Alarmingly, it is estimated that 90% of rare diseases lack approved therapies [8]. Furthermore, the unique nature of rare diseases often isolates patients and their families, forcing them to navigate complex and fragmented healthcare systems with minimal support.

In the Middle East, challenges related to rare diseases are further exacerbated by region-specific factors, most notably high rates of consanguinity, which significantly increase the prevalence of genetic disorders and the lack of comprehensive country-wide registries, which limits the availability of epidemiological data and patient advocacy efforts [3, 9–11]. Sociocultural barriers, such as stigma and limited public awareness, along with fragmented healthcare systems lacking specialized providers, infrastructure, and resources, further complicate patient care [3, 9]. Several studies emphasize the importance of epidemiological insights in bridging knowledge gaps and supporting patient advocacy to tackle these issues effectively [4, 12]. Insufficient registries, limited diagnostic capabilities, and inequities in access to care further highlight the pressing need for coordinated efforts to improve healthcare systems and promote cross-sector collaboration.

Recognizing these urgent needs, the Rare Advocacy Council brought together experts from multiple countries across the Middle East, along with international specialists, to address regional issues related to rare diseases.

This council adopted a multidisciplinary approach to rare disease advocacy and policymaking to address the key challenges confronting patients with rare diseases in the Middle East and propose actionable strategies to enhance healthcare delivery and advocate for policy reforms. The ultimate goal is to ensure fair practices and equitable access to care, thereby improving the quality of life for patients with rare diseases and their families while establishing a sustainable framework for managing rare diseases in the region.

## Methods

**Fig. 1:**
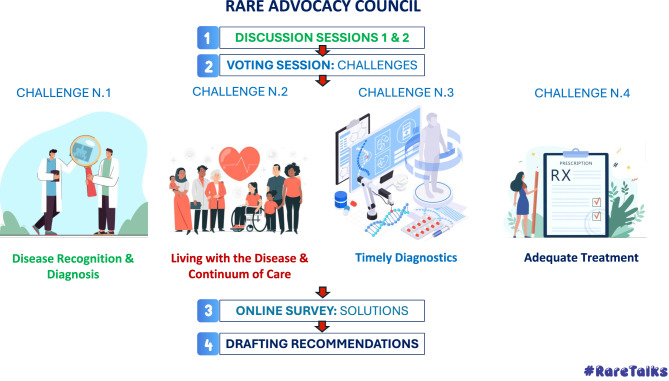
Rare Advocacy Council project stages

The Rare Disease Advocacy Council brought together a multidisciplinary panel of experts from several countries across the Middle East alongside international experts to address region-specific challenges related to rare diseases through a series of 1.5-hour virtual events. This study employed a multi-phase approach to identify and prioritize challenges, ultimately leading to actionable recommendations (Fig. 1).

### Participants and panel composition

A total of 16 participants took part in the initiative. Panel members were purposively selected based on their recognized expertise and active involvement in rare disease care, advocacy, research, or policy-related activities. The panel included clinicians (*n* = 5), patient advocates (*n* = 4), and international academic experts (*n* = 5). Industry representatives (*n* = 2) attended in a strictly observational, non-voting capacity and did not participate in the discussions or influence the content, priorities, or recommendations. They had no role in directing the work and did not influence the study design, data interpretation, or manuscript content. Clinical expertise covered key specialties relevant to rare diseases, including hematology-oncology, pediatric hematology-oncology, and cardiology. International academic experts contributed by sharing their experiences in addressing comparable challenges within their respective regions. Participants represented multiple countries across the Middle East region (United Arab Emirates, Kingdom of Saudi Arabia, Bahrain, Qatar, and Oman) and Europe (France, Belgium, the Netherlands, Serbia, and Romania), ensuring both regional relevance and international perspective (Supplementary Material 1).

Inclusion criteria for participation included: (1) demonstrated professional experience in rare diseases; (2) active involvement in clinical care, patient advocacy, research, or health system decision-making; and (3) familiarity with regional healthcare systems and policy environments. Patients themselves were represented through patient advocates actively engaged in organized patient groups.

### Structure of the virtual sessions

Two discussion sessions explored challenges across four key domains: (1) *Disease recognition and diagnosis*; (2) *Living with rare diseases and the continuum of care*; (3) *Access to timely diagnostics*; and (4) *Access to adequate treatment*. Each session followed a standardized three-step structure, including identification of domain-specific challenges, expert-led discussion to contextualize these challenges from clinical, patient, and health system perspectives, and the development of actionable, regionally relevant solutions.

### Prioritization and voting process

Following the discussion sessions, only regional clinicians and patient advocates (*n* = 9) engaged in a structured online voting exercise, as the objective was to capture challenges specific to the region. Challenges identified within each domain were rated using a 5-point Likert scale: *Strongly Significant*, *Significant*, *Neutral*, *Insignificant*, or *Strongly Insignificant*. They were then asked to independently answer a prioritization question tailored to decision-making contexts: “When presenting the challenges of disease recognition and diagnosis to a decision-maker or payer, which challenge, in your opinion, should be addressed as the top priority?“. The options most frequently selected by the panel experts were used as the basis for the next step: the *follow-up survey and the development of recommendations*.

Prioritization therefore reflected experts’ individual judgments regarding policy relevance and feasibility. Results were analyzed descriptively based on Likert-scale ratings and frequency of priority selections.

### Follow-up survey and development of recommendations

A subsequent online survey was conducted to collect proposed solutions to the priority challenges identified during the voting phase. Survey questions were directly informed by the issues raised and discussions held during each virtual session. Detailed information on the questions addressed within each domain is provided in Supplementary Material 1. The survey was distributed to fourteen stakeholders, including international experts, but excluding the industry representatives, to gather their insights on the most appropriate and contextually adapted solutions for implementing, drawing on both local needs and international experience. Twelve participants completed the survey (12/14; response rate: 85.7%), despite two email reminders and two phone reminders. The two non-respondents were international experts; all experts from the Middle East participated in the survey.

Responses were thematically analyzed to synthesize actionable recommendations.

The final step involved summarizing findings, drafting recommendations, and formulating an action plan to engage policymakers and healthcare providers in addressing these critical issues. Each event focused on one of the main challenge areas.

## Results

### *Section 1*: Challenges of disease recognition and diagnosis

**Fig. 2:**
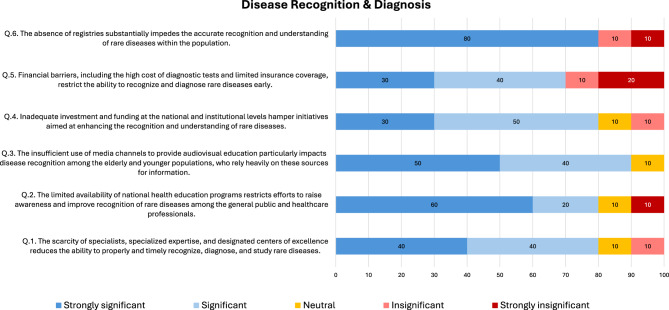
Voting results on challenges in disease recognition and diagnosis identified by the Rare Advocacy Council (in percentages; *n* = 9)

**Challenge 1.1.** The absence of registries substantially impedes the accurate recognition and understanding of rare diseases within the population (80% rated the challenge as strongly significant, with 40% identifying it as the top priority to be presented to a decision-maker or payer; Fig. 2, Q.6.).

**Challenge 1.2.** The insufficient use of media channels to provide audiovisual education particularly impacts disease recognition among the elderly and younger populations, who rely heavily on these sources for information (70% rated the challenge as strongly significant or significant, with 20% identifying it as the top priority to be presented to a decision-maker or payer; Fig. 2, Q.3.).

**Fig. 3:**
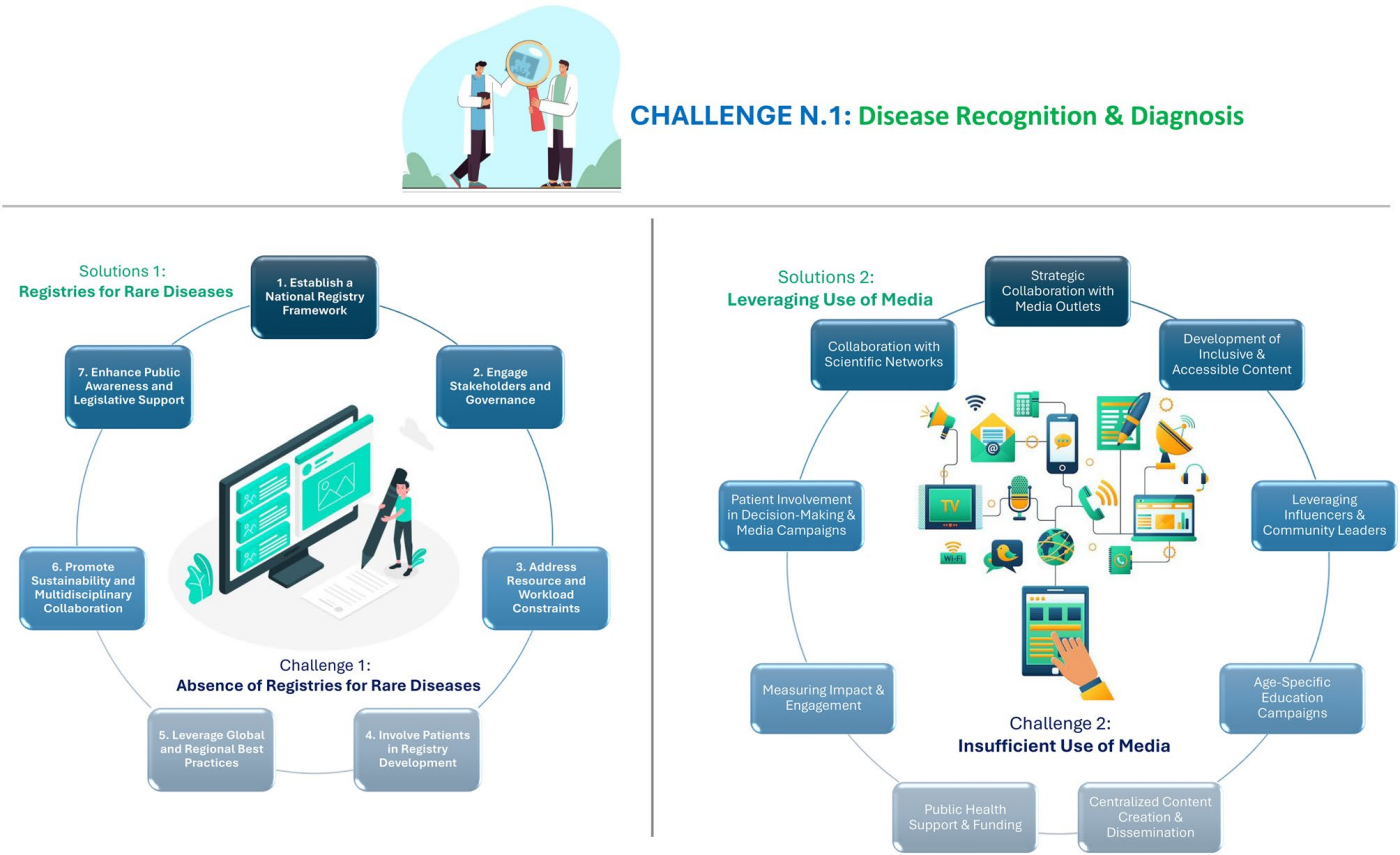
Top two prioritized challenges and proposed solutions for “Disease recognition and Diagnosis” based on participant voting

**Thematic solutions to address the absence of registries for rare diseases: Challenge n.1.1 **(Fig. 3a).

**1. Policy and governance**

- Assign Ministry of Health (MOH) to leadership to drive registry initiatives.
- Explore partnerships with pharmaceutical companies and hospital for support.

**2. Capacity building and infrastructure**

- Initiate pilot registries as models for broader implementation.
- Create centralized, standardized database integrating data from multiple healthcare providers, in collaboration and partnerships with patient advocacy groups (PAGs) and relevant societies.
- Form dedicated teams of clinicians and statisticians to create, maintain and advocate for databases and registries.

**3. Patients empowerment**

- Enable patient access to their registries data.
- Incorporate Patient-Reported Outcomes (PROs), such as Quality of Life (QoL) data, to enhance the registry’s value.
- Engage patient advocacy groups in registry development and promotion.

**4. Knowledge sharing and sustainability**

- Adopt best practices from successful registry models, such as the French National Registry for Rare Diseases (https://www.bndmr.fr/), or the European Reference Networks (ERNs) (https://eurreb.eu/).
- Implement a multidisciplinary approach that involves healthcare professionals across different fields, including geneticists, statisticians, and clinicians, to ensure comprehensive patient care and robust registry development.
- Raise community awareness about rare diseases through accessible educational tools to enhance recognition and data reporting.

The complete list of the proposed solutions to address the absence of registries are illustrated in Fig. 3 and can be found in Supplementary Material 2.

**Thematic solutions to address the insufficient use of media: Challenge n.1.2 **(Fig. 3b).

**1. Policy and governance**

- Advocate for public health funding to create professional, high-quality audiovisual campaigns.
- Promote partnerships with educational institutions and pharmaceutical companies to develop materials and produce content that raise awareness about rare diseases.
- Collaborate with organizations such as Endo-ERN, which works with societies like the European Society for Endocrinology (ESE) and the European Society for Paediatric Endocrinology (ESPE), allowing for more targeted educational efforts.

**2. Capacity building and infrastructure**

- Establish a centralized team involving public health authorities, patient advocacy groups, and media experts to design consistent and unified awareness campaigns.
- Create audiovisual materials with simplified language, visuals, and accessibility features (e.g., subtitles, audio descriptions, and sign language).

**3. Patient empowerment**

- Involve patients more actively in decision-making processes related to media campaigns. Having patients share their personal experiences helps humanize the disease and ensures a more relatable message.
- Use storytelling techniques and real-life patient testimonials to make the content relatable and emotionally engaging.

**4. Public engagement and communication strategies**

- Partner with mainstream media (TV, radio, and newspapers) to produce educational campaigns focused on rare diseases tailored to different demographics.
- Target younger populations through short, visually appealing videos, using digital platforms like YouTube, TikTok, and Instagram.
- Design age-specific campaigns: interactive content for younger audiences; traditional media for older populations.
- Measure impact using analytics, surveys, or focus groups to refine campaigns.

More details are described in Fig. 3 and Supplementary Material 2.

### *Section 2*: Challenges of living with the disease & the continuum of care

**Fig. 4:**
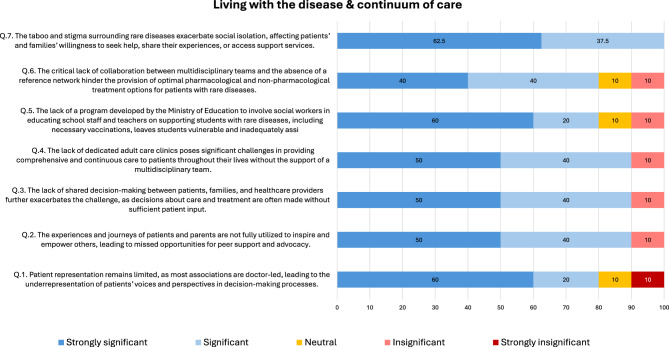
Voting results on living with the disease and continuum of care identified by the Rare Advocacy Council (in percentages; *n* = 9)

Challenge “Q.7. *The taboo and stigma surrounding rare diseases exacerbate social isolation*,* affecting patients’ and families’ willingness to seek help*,* share their experiences*,* or access support services*” was unanimously identified by all participants (100%) as the most critical challenge for patients living with these conditions and for the continuum of care. However, as this challenge is not directly actionable for stakeholders, two other challenges, those most frequently selected by the panel as top priorities, were selected as key issues to address and consider when proposing solutions (Fig. 4).

**Challenge 2.1.** Patient representation remains limited, as most associations are doctor-led, leading to the underrepresentation of patients’ voices and perspectives in decision-making processes (80% strongly significant or significant; 50% top priority to be presented to a decision-maker or payer; Fig. 4, Q.1.).

**Challenge 2.2.** The critical lack of collaboration between multidisciplinary teams and the absence of a reference network hinder the provision of optimal pharmacological and non-pharmacological treatment options for patients with rare diseases (80% strongly significant or significant; 20% top priority to be presented to a decision-maker or payer; Fig. 4, Q.6.).

**Fig. 5:**
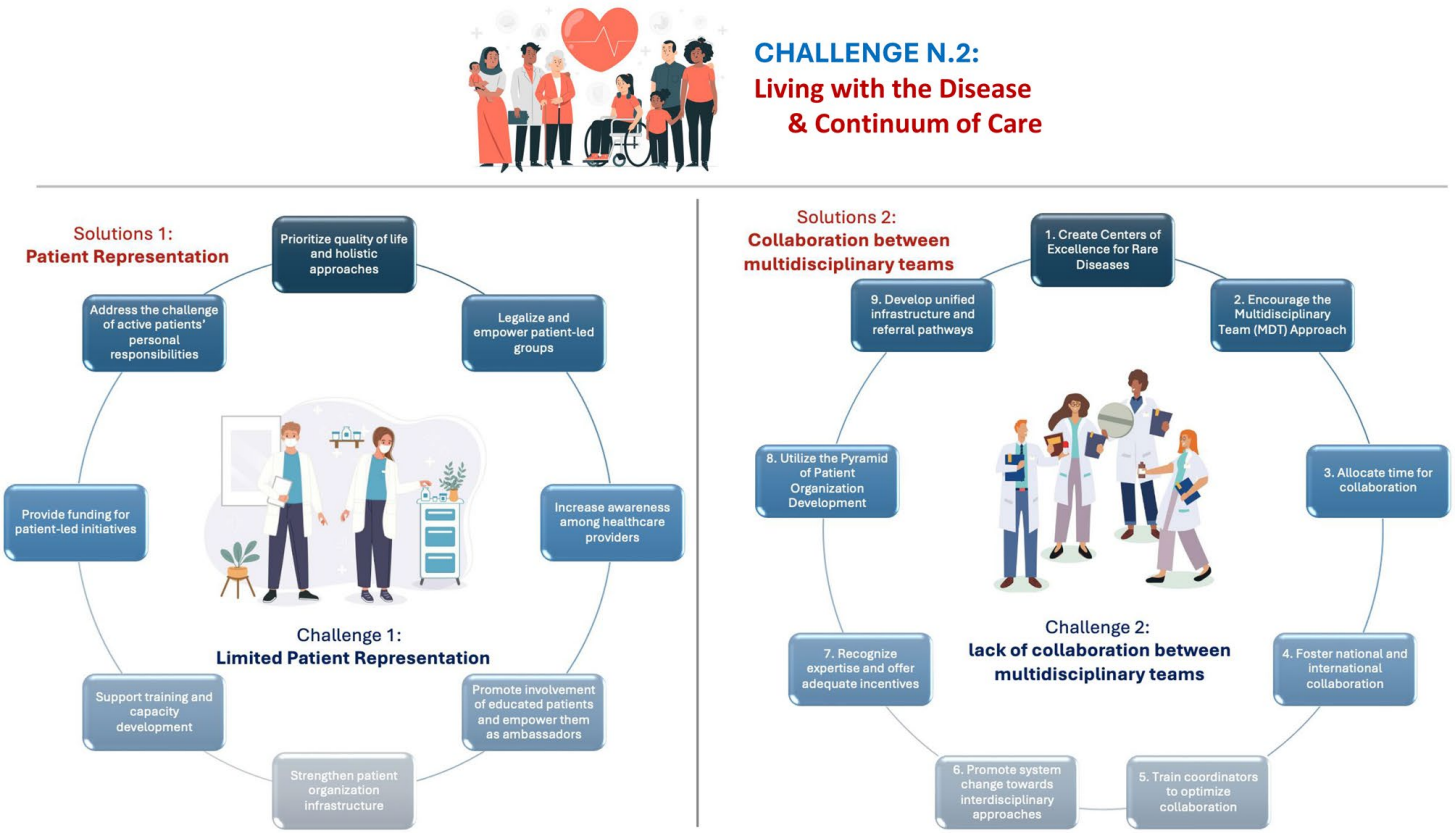
Top two prioritized challenges and proposed solutions for “Living with the disease & the Continuum of care” based on participant voting

**Thematic solutions to address limited patient representation: Challenge n.2.1 **(Fig. 5a).

**1. Policy and governance**

- Legalize and empower patient-led groups to ensure the patient’s voices are properly represented in decision-making processes.
- Strengthen collaboration among ministries (health, education, and social services) to better address the diverse needs of patients with rare diseases.
- Promote care models that explicitly prioritize quality of life alongside medical management.

**2. Capacity building**

- Provide structured training and capacity-development programs for patient organizations to strengthen their capabilities to advocate and collaborate effectively with healthcare professionals.
- Improve care providers’ understanding and increase doctors’ awareness of the importance of patient partnership and engagement in care decisions.
- Organize discussions between doctors and patient representatives to foster mutual understanding of the benefits of collaboration, including engagement with international experts.

**3. Patient empowerment**

- Identify and empower some key patients with rare diseases to act as ambassadors in patient groups and advocacy campaigns.
- Select young patients to be representatives, as they are often more motivated and equipped to engage in advocacy and awareness campaigns. For example, in 2012, a PhD patient with amyloidosis began leading a patient group and became an ambassador, raising awareness and educating others about treatment options and disease understanding.
- Encourage doctors to assist active patients in founding patient organizations, which can be critical in helping with information dissemination and advocacy.

**4. Infrastructure, funding, and sustainability**

- Strengthen patient organization infrastructure that hinders collaboration. For example, in 1996, doctors helped active patients by providing them with information to create leaflets and website content and speaking at patient meetings.
- Acknowledge the challenges faced by active patients and their families, who may be balancing advocacy work with personal responsibilities such as caring for other children or managing their health.
- Address the issue of funding patient-led initiatives by seeking grants and organizational support from healthcare institutions and charities to help cover expenses for events, meetings, and awareness campaigns.

Figure 5 and Supplementary Material 2 provide a detailed overview of these solutions.

**Thematic solutions to address lack of collaboration between multidisciplinary teams: Challenge n.2.2 **(Fig. 5b).

**1. Policy and governance**

- Implement a multidisciplinary approach for rare disease management that ensures comprehensive care, with multidisciplinary teams (MDTs) established within specialized centers and academic hospitals, which offer a collaborative environment for various specialties.
- Establish regional or national centers of excellence for rare diseases that centralize expertise and resources, modeled after successful examples, such as pituitary centers of excellence where integrated MDTs deliver coordinated, expert patient care.
- Recognize MDT-based care as a standard of practice and align institutional policies accordingly.

**2. Capacity building**

- Allocate protected time for clinicians to meet and collaborate, ensuring that MDT meetings are not compromised by competing clinical schedules.
- Train coordinators to optimize collaboration within and between MDTs, improving communication and scheduling.
- Support continuous professional development through international collaborations, congress participation, and mentorship programs.

**3. Care delivery models**

- Advocate for a system change to institutionalize the interdisciplinary approach for rare diseases, ensuring policies support MDT operations in centers of expertise.
- Highlight and promote successful models of MDT approaches, such as the pituitary centers of excellence, to demonstrate their impact on patient outcomes and care coordination.

**4. Infrastructure, funding, and sustainability**

- Develop well-defined referral pathways between public and private sectors to ensure timely access to specialized care and improve coordination among healthcare providers.
- Foster national and international collaboration through scientific networks and expert partnerships.
- Recognize and reward expertise by ensuring adequate compensation for professionals working within interdisciplinary teams.

More details are described in Fig. 5 and Supplementary Material 2.

### *Section 3*: Challenges of accessing timely diagnostics

**Fig. 6:**
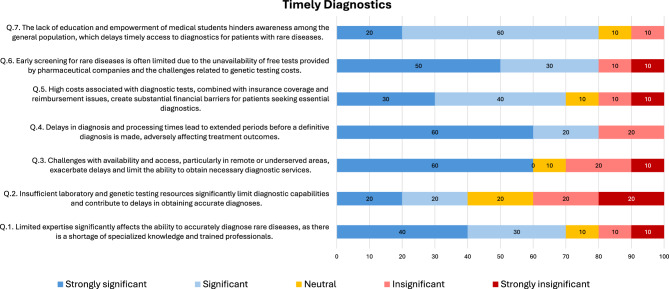
Voting results on timely diagnostics identified by the Rare Advocacy Council (in percentages; *n* = 9)

**Challenge 3.1.** Limited expertise hinders the accurate diagnosis of rare diseases due to a shortage of specialized knowledge and trained professionals (70% highly significant or significant; 40% top priority to be presented to a decision-maker or payer) (Fig. 6, Q.1.).

**Challenge 3.2.** Early screening for rare diseases is often constrained by the lack of free tests provided by pharmaceutical companies and the high costs associated with genetic testing (80% highly significant or significant; 20% top priority to be presented to a decision-maker or payer) (Fig. 6, Q.6.).

**Fig. 7:**
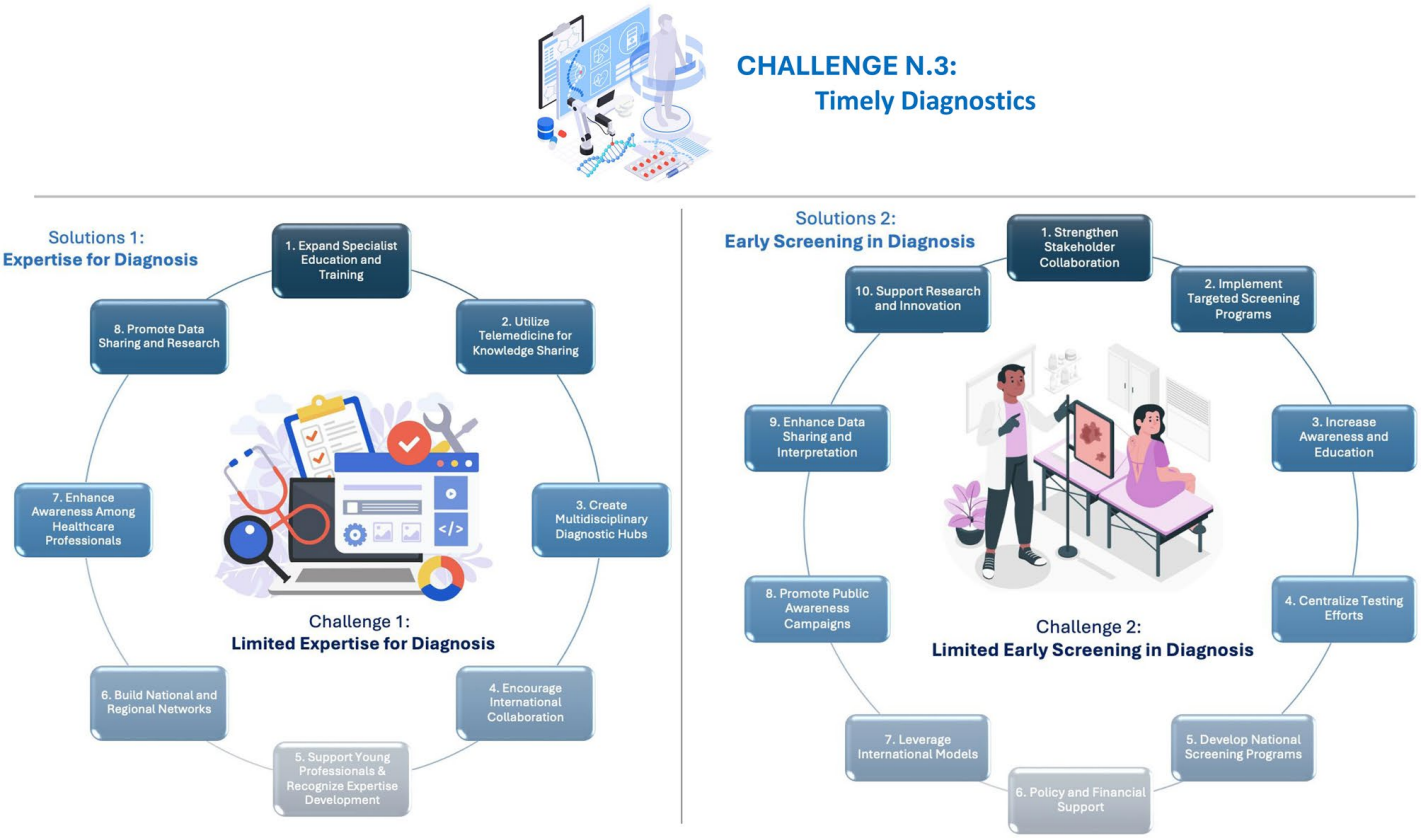
Top two prioritized challenges and proposed solutions for “Accessing timely diagnostics” based on participant voting

**Thematic solutions to address limited expertise for diagnosis: Challenge n.3.1 **(Fig. 7a).

**1. Policy and governance**

- Support the creation of regional and national diagnostic centers of excellence with sustainable funding.
- Promote formal recognition, certification, and career advancement pathways for healthcare providers specializing in rare diseases.

**2. Capacity building**

- Develop residency, fellowship, and subspecialty programs focused on rare diseases.
- Provide financial incentives, such as scholarships or grants, to encourage healthcare professionals to specialize in rare diseases.
- Establish mentorship programs where experienced specialists in rare diseases guide young professionals.
- Integrate rare disease topics into medical school curricula and continuing medical education (CME) programs.

**3. Care delivery models**

- Develop regional centers of excellence with dedicated MDT, including geneticists, bioinformaticians, and biochemical specialists.
- Implement telemedicine platforms to connect local practitioners with experts in rare diseases for real-time consultations and case discussions.
- Organize regular grand rounds, case reviews, and virtual conferences focused on rare disease diagnostics.

**4. Collaboration & networks**

- Establish partnerships with global centers of excellence to facilitate exchange programs, observerships, and access to cutting-edge expertise.
- Encourage participation in international congresses and global expert networks.
- Facilitate regular discussions and case reviews among healthcare providers through online networks and collaborative platforms.

**5. Data infrastructure & research**

- Create a centralized directory of rare disease specialists and diagnostic facilities for streamlined referrals.
- Collaborate with research institutions to study rare disease patterns and identify biomarkers for improved diagnosis.
- Promote integration of clinical and research data to accelerate expertise development.

More details are provided in Fig. 7 and Supplementary material 1.

**Thematic solutions to address limited early screening in diagnosis: Challenge n.3.2 **(Fig. 7b).

**1. Policy and governance**

- Advocate for national frameworks supporting genetic testing, including newborn and premarital screening programs.
- Engage governments, insurers, and policymakers to ensure reimbursement and long-term sustainability of screening initiatives.
- Highlight the health and economic benefits of early screening to inform policy and payer decisions.

**2. Capacity building**

- Educate healthcare professionals, including nurses and physicians, on the availability and processes of free genetic tests.

**3. Screening infrastructure**

- Establish regional centers for rare disease screening to consolidate resources and expertise.
- Integrate telemedicine platforms for test result analysis and expert consultations.
- Develop targeted pilot screening programs focused on high-prevalence or actionable rare diseases.

**4. Stakeholder engagement & collaboration**

- Strengthen partnerships with pharmaceutical companies to expand free or subsidized genetic testing programs.
- Foster long-term collaboration with international networks to exchange best practices and technical expertise.
- Engage insurance providers early to align coverage policies with screening strategies.

**5. Public awareness & social acceptance**

- Conduct awareness initiatives to address cultural and social barriers to genetic testing, such as those related to consanguinity.
- Emphasize the importance of early screening in reducing the burden of rare diseases.
- Develop workshops and materials to improve the efficiency of test administration in hospitals.

**6. Data infrastructure, research & innovation**

- Develop centralized databases for genetic testing results to improve pattern recognition and rare disease diagnostics.
- Encourage research on cost-effective and targeted genetic testing approaches.
- Partner with pharmaceutical companies to fund the development of innovative diagnostic tools (Fig. 7 & Supplementary Material 2).

### *Section 4*: Challenges of access to adequate treatment

**Fig. 8:**
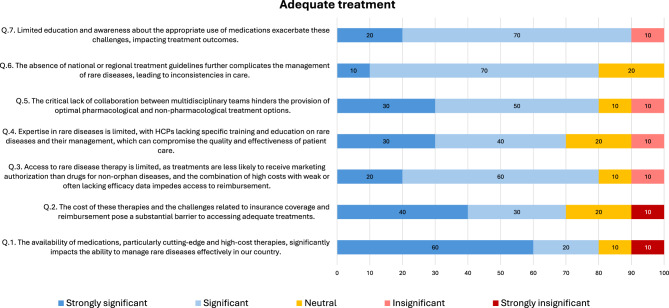
Voting results on adequate treatment identified by the Rare Advocacy Council (in percentages; *n* = 9)

**Challenge 4.1.** The limited availability of medications, particularly cutting-edge and high-cost therapies, significantly hampers effective rare disease management in our country (80% highly significant or significant; 40% top priority for presentation to a decision-maker or payer) (Fig. 8; Q.1.).

**Challenge 4.2.** The high cost of these therapies and the challenges related to insurance coverage and reimbursement pose a substantial barrier to accessing adequate treatment (70% highly significant or significant; 20% top priority to be presented to a decision-maker or payer) (Fig. 8, Q.2.).

**Fig. 9:**
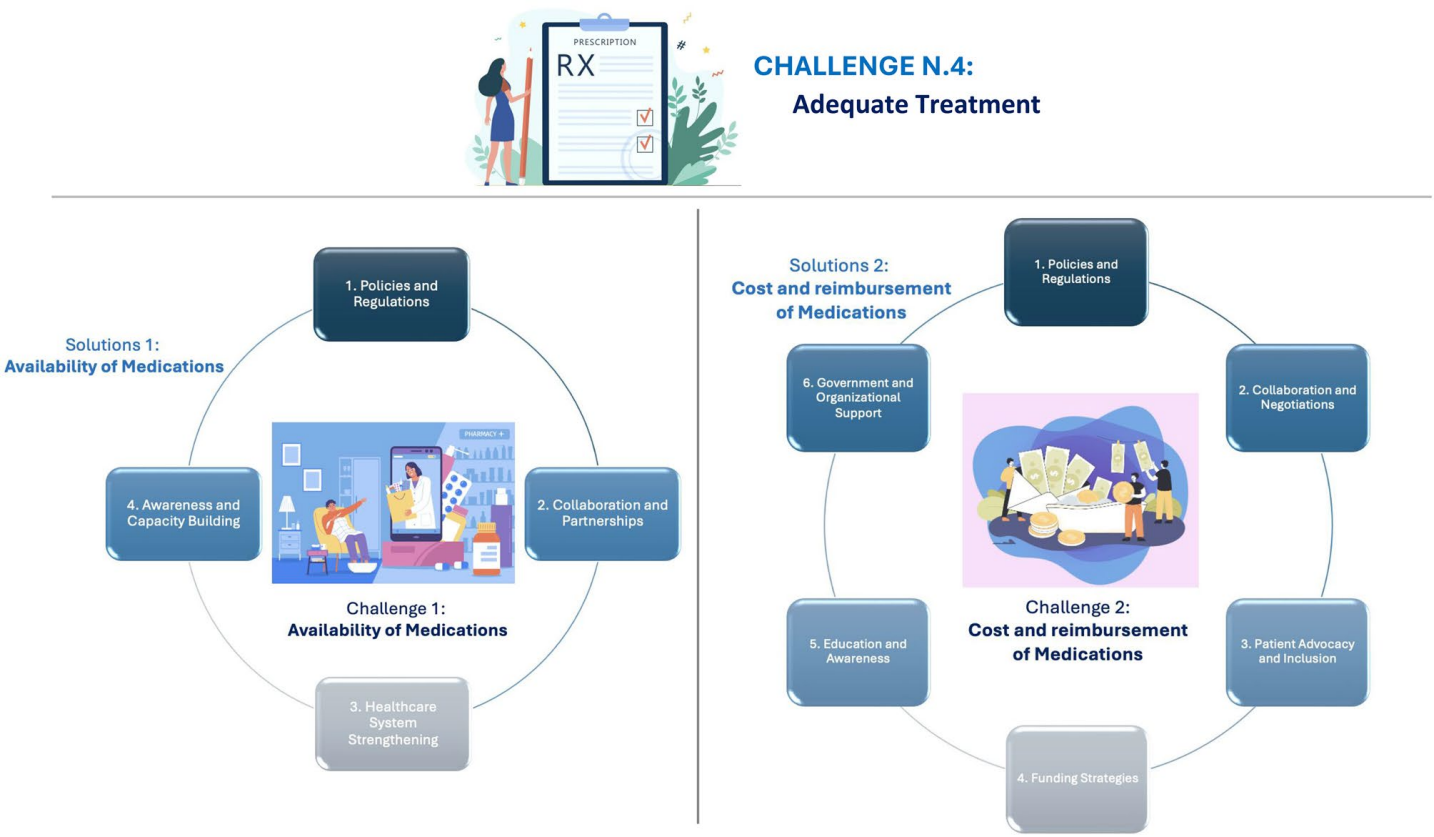
Top two prioritized challenges and proposed solutions for “Access to adequate treatment” based on participant voting

**Thematic solutions to availability of medications: Challenge n.4.1 **(Fig. 9a).

**1. Policies and governance**

- Streamline and expedite approval processes for orphan drugs to avoid delays in their market availability.
- Encourage governments to collaborate with academic and private institutions to share the costs and risks of developing expensive therapies.

**2. Collaboration and partnerships**

- Support clinical trials and research collaborations in strategic regions, such as the United Arab Emirates, to introduce innovative therapies.
- Foster international collaborations to share resources and financial burdens associated with managing rare diseases.

**3. Healthcare system strengthening**

- Promote balanced pharmaceutical strategies that ensure access to both innovative therapies and essential, affordable generic medications.
- Encourage pharmaceutical companies marketing high-cost treatments to also maintain the supply of low-cost essential medicines.
- Ensure comprehensive medication availability to address multimorbidity commonly experienced by patients with rare diseases.

**4. Awareness and capacity building**

- Increase awareness among healthcare providers about rare disease treatments and their importance in enhancing early diagnosis and management.
- Organize training programs and promote knowledge sharing to ensure effective use of available resources.

**Thematic solutions to cost and reimbursement of medications: Challenge n.4.2 **(Fig. 9b).

**1. Policies and governance**

- Advocate for policy adaptations requiring acceptable coverage of life-saving therapies by health insurance companies.
- Expand formulary approvals to include essential therapies for rare diseases.
- Introduce government-backed initiatives and international collaborations to facilitate affordable access to treatments for rare diseases, particularly in low- and middle-income settings.
- Reevaluate pricing structures to better reflect regional economic realities and patient needs.

**2. Collaboration and negotiation**

- Facilitate direct negotiations between payers (government or private) and manufacturers to achieve fair pricing.
- Promote regional collaboration in Health Technology Assessments (HTA) to ensure consistent decisions across neighboring countries.
- Encourage partnerships between pharmaceutical companies and charitable organizations to support access programs for patients in need.

**3. Patient advocacy and inclusion**

- Leverage the patient voice as a negotiation tool with insurance companies and policymakers to emphasize the importance of access to therapies.
- Advocate for mandatory patient involvement in HTA decisions and regulatory processes to ensure patient needs and quality of life enhancements are prioritized.
- Support programs that provide funding for patients who cannot afford their medications and offer opportunities for others to receive free medications.

**4. Funding strategies**

- Develop pricing models that link drug costs to their corresponding health outcomes, enabling patients to pay based on the effectiveness of their treatment.
- Encourage pharmaceutical companies to lower prices for low-income countries or offer sponsorship programs.
- Implement funding strategies that support equal access to both high-cost and essential therapies for patients with rare diseases.

**5. Education and awareness**

- Educate the public about the appeal process for insurance claims related to rare disease treatments.
- Enhance awareness among decision-makers about the unmet needs of patients and the value of new therapies in improving quality of life.
- Promote awareness of programs and initiatives that enable patients to access necessary treatments, including awareness about insurance claims processes and patient assistance programs.

**6. Government and organizational support**

- Advocate for government-sponsored coverage in countries where the cost of therapies is prohibitive.
- Use concerned medical committees to lobby for better insurance regulations and improved reimbursement frameworks.
- Foster international collaboration to reduce costs and share resources, ensuring that patients in all regions can access necessary therapies.

Solutions for the main two challenges are illustrated in Fig. 9 and Supplementary Material 2.

## Discussion and conclusion

Rare diseases present a complex set of challenges that require innovative and collaborative solutions. This white paper from the Rare Advocacy Council has identified critical barriers across the Middle East region, including inadequate registry infrastructure, insufficient patient representation, fragmented multidisciplinary care, particularly limited coordination among providers, and persistent diagnostic and treatment delays. These findings reflect interconnected health system constraints that affect the entire rare disease care continuum.

Our findings align with global rare-disease initiatives such as the WHO Resolution on Rare Diseases (2025) [13], the International Rare Diseases Research Consortium (IRDiRC) Vision and Goals 2027 [14], EURORDIS advocacy frameworks [15], and the European Reference Networks (ERNs) policy framework. They also resonate with national rare-disease plans, such as France’s Plan Maladies Rares 3 [16] and the UAE’s national strategy. However, regional differences in healthcare systems, resources, and infrastructure within the Middle East affect the feasibility of implementing similar initiatives. For example, while establishing centers of excellence may be feasible in Gulf Cooperation Council (GCC) countries with centralized healthcare funding, resource-limited settings may prioritize strengthening registries and patient networks first.

Addressing these challenges requires investment in foundational infrastructure, particularly comprehensive national registries, to improve disease recognition, enable epidemiological analyses, and support targeted interventions.

In parallel, insufficient public and professional awareness contributes to delayed diagnosis and limited patient engagement, highlighting the potential role of strategic media partnerships. The analysis also underscored the importance of specialized centers of excellence in concentrating expertise and addressing the complex care needs of people living with rare diseases. Together, these elements illustrate how structural and organizational factors shape patient outcomes beyond individual clinical visits. Additionally, strategic media partnerships can substantially raise public awareness, while creating specialized centers of excellence can deliver expertise for complex care needs. Concurrent policy advocacy remains essential for improving treatment accessibility and affordability.

From a health system and policy perspective, the findings suggest that rare disease initiatives are most effective when embedded within broader national health frameworks. Each stakeholder bears distinct responsibilities in advancing the rare disease agenda. Policymakers must prioritize rare diseases within national health frameworks, allocating sufficient resources for research and enacting supportive policies. Healthcare providers should contribute by adopting a multidisciplinary approaches and commit engaging into continuous professional development to enhance improve diagnosis accuracy and patient care coordination. Advocacy groups are uniquely positioned to amplify patient voices, collect critical data, and collaborate with stakeholders, thus informing evidence-based policies and driving impactful changes.

This work has several limitations. First, the findings are based on expert opinion derived from structured discussions and descriptive analyses, rather than quantitative outcome measures. Although the panel included diverse professional profiles and regional representation, the number of participants was limited, thus introducing potential bias. Finally, regional heterogeneity across the different countries from the Middle East limits generalizability of recommendations. As such, the results should be interpreted as an exploratory synthesis of stakeholder perspectives rather than a comprehensive evaluation of national systems.

In conclusion, advancing rare disease research and improving patient outcomes require sustained collaborative efforts. Critical next steps include expanding research initiatives, fostering international partnerships aligned with global frameworks (e.g., IRDiRC, ERNs, EURORDIS), and developing comprehensive care models that address rare disease patients’ complex needs. Shared research infrastructures, active patient engagement across the research process, and enhanced stakeholder coordination are the foundation for sustainable progress. Furthermore, continuous evaluation mechanisms will ensure strategy adaptation and refinement over time. By aligning the efforts of policymakers, healthcare providers, researchers, and advocacy groups, it is possible to create a future in which rare disease patients receive the care, recognition, and support they need and deserve.

## Supplementary Information

Below is the link to the electronic supplementary material.

Supplementary Material 1

Supplementary Material 2

## Data Availability

The datasets used and/or analyzed during the current study are available from the corresponding author on reasonable request.

## Abbreviations

CME: Continuing medical education
ERNs: European Reference Networks
ESE: European Society for Endocrinology
ESPE: European Society for Paediatric Endocrinology
GCC: Gulf Cooperation Council
HTA: Health Technology Assessments
IRDiRC: International Rare Diseases Research Consortium
MDTs: Multidisciplinary Teams
MOH: Ministry of Health
PAGs: Patient Advocacy Groups
PROs: Patient-Reported Outcomes
QoL: Quality of Life
