# Supplementary material for: Management of patients with rare diseases in the Middle East: challenges & opportunities – insights from the Rare Advocacy Council
Source: Orphanet J Rare Dis. 2026 Jul 31;21:260. doi: 10.1186/s13023-026-04469-1 (PMC13428422; doi:10.1186/s13023-026-04469-1)
Supplement: Supplementary file 1 — Supplementary Material 1 [file 13023_2026_4469_MOESM1_ESM.docx]

**SUPPLEMENTARY MATERIAL 1**

**Supplementary Table 1:** Panel composition by country, stakeholder type, and participation stage.

| **Country** | **Stakeholder Type** | **Participation Stage** |
| --- | --- | --- |
| Qatar | Patient advocates | Sessions/voting/survey |
| UAE 2 | Patient advocates | Sessions/voting/survey |
| Bahrain | Patient advocates | Sessions/voting/survey |
| Saudi Arabia 4 | Clinicians | Sessions/voting/survey |
| Oman | Clinicians | Sessions/voting/survey |
| Serbia | International academic experts | Sessions/survey |
| The Netherlands | International academic experts | Sessions/survey |
| Romania | International academic experts | Sessions/survey |
| Belgium | International academic experts | Sessions/survey |
| France | International academic experts | Sessions/survey |
| Sydney | Industry representatives | Sessions: support role |
| Egypt | Industry representatives | Sessions: support role |
| Lebanon | Science PRO | Sessions: support role |

**SECTION 1: CHALLENGES OF DISEASE RECOGNITION & DIAGNOSIS**

**Rate the significance of the below challenges of DISEASE RECOGNITION & DIAGNOSIS for individuals with rare diseases in your country**

Q.1. The scarcity of specialists, specialized expertise, and designated centers of excellence reduces the ability to properly and timely recognize, diagnose, and study rare diseases.

Q.2. The limited availability of national health education programs restricts efforts to raise awareness and improve recognition of rare diseases among the general public and healthcare professionals.

Q.3. The insufficient use of media channels to provide audiovisual education particularly impacts disease recognition among the elderly and younger populations, who rely heavily on these sources for information.

Q.4. Inadequate investment and funding at the national and institutional levels hamper initiatives aimed at enhancing the recognition and understanding of rare diseases.

Q.5. Financial barriers, including the high cost of diagnostic tests and limited insurance coverage, restrict the ability to recognize and diagnose rare diseases early.

Q.6. The absence of registries substantially impedes the accurate recognition and understanding of rare diseases within the population.

Q.7. Are there any additional challenges related to disease recognition and diagnosis of rare diseases that have not been covered in our discussion?

- The absence of clinical trials for rare diseases in our region reduces the awareness of clinical experience among healthcare providers.
- Each part of the world faces different types of diseases and chronic illnesses. We found that medical schools are disconnected from community challenges and tend to copy other nations’ materials without considering national diseases, making education among caregivers highly challenging.
- Increasing the knowledge of primary health doctors is essential.
- There is a lack of knowledge among general practitioners (GPs) regarding rare diseases, and improper referrals are common.
- There is a lack of awareness about rare diseases among healthcare professionals.

**When presenting the challenges of disease recognition and diagnosis to a decision-maker or payer, which challenge, in your opinion, should be addressed as the top priority?**

- **Q.6. (n=40%):** The absence of registries substantially impedes the accurate recognition and understanding of rare diseases within the population
- **Q.3. (n=20%):** The insufficient use of media channels to provide audiovisual education particularly impacts disease recognition among the elderly and younger populations, who rely heavily on these sources for information
- **Q.2. (n=20%):** The limited availability of national health education programs restricts efforts to raise awareness and improve recognition of rare diseases among the general public and healthcare professionals
- **Q.1. (n=20%):** The scarcity of specialists, specialized expertise, and designated centers of excellence reduces the ability to properly and timely recognize, diagnose, and study rare diseases

**SECTION 2: CHALLENGES OF LIVING WITH THE DISEASE & THE CONTINUUM OF CARE**

**Rate the significance of the below challenges of LIVING WITH A DISEASE & THE CONTINUUM OF CARE for individuals with rare diseases in your country**

Q.1. Patient representation remains limited, as most associations are doctor-led, leading to the underrepresentation of patients’ voices and perspectives in decision-making processes.

Q.2. The experiences and journeys of patients and parents are not fully utilized to inspire and empower others, leading to missed opportunities for peer support and advocacy.

Q.3. The lack of shared decision-making between patients, families, and healthcare providers further exacerbates the challenge, as decisions about care and treatment are often made without sufficient patient input.

Q.4. The lack of dedicated adult care clinics poses significant challenges in providing comprehensive and continuous care to patients throughout their lives without the support of a multidisciplinary team.

Q.5. The lack of a program developed by the Ministry of Education to involve social workers in educating school staff and teachers on supporting students with rare diseases, including necessary vaccinations, leaves students vulnerable and inadequately assisted.

Q.6. The critical lack of collaboration between multidisciplinary teams and the absence of a reference network hinder the provision of optimal pharmacological and non-pharmacological treatment options for patients with rare diseases.

Q.7. The taboo and stigma surrounding rare diseases exacerbate social isolation, affecting patients’ and families’ willingness to seek help, share their experiences, or access support services.

Q.8. Are there any additional challenges related to living with the disease and the continuum of care for rare diseases that have not been covered in our discussion?

- Challenges in insurance coverage arise as patients move into advanced stages of the disease, requiring more tests, procedures, therapies, devices, supplements, etc.
- Disabilities caused by the disease and the lack of regulations add more challenges for individuals living with their illnesses.
- Financial challenges are significant, especially for many poor families.
- There is a need for more doctors in peripheral hospitals and better networking with their Medical Responsible Physicians (MRPs).
- There is a lack of occupational programs available.
- Social stigma and insufficient awareness lead some families to delay presenting their abnormal child to the doctor, which postpones early diagnosis and treatment.

**When presenting the challenges of disease recognition and diagnosis to a decision-maker or payer, which challenge, in your opinion, should be addressed as the top priority?**

- **Q.1. (n=50%):** Patient representation remains limited, as most associations are doctor-led, leading to the underrepresentation of patients’ voices and perspectives in decision-making processes.
- **Q.6. (n=20%):** The critical lack of collaboration between multidisciplinary teams and the absence of a reference network hinder the provision of optimal pharmacological and non-pharmacological treatment options for patients with rare diseases.
- **Q.2. (n=10%):** The experiences and journeys of patients and parents are not fully utilized to inspire and empower others, leading to missed opportunities for peer support and advocacy.
- **Q.4. (n=10%):** The lack of dedicated adult care clinics poses significant challenges in providing comprehensive and continuous care to patients throughout their lives without the support of a multidisciplinary team.
- **Q.3. (n=10%):** The lack of shared decision-making between patients, families, and healthcare providers further exacerbates the challenge, as decisions about care and treatment are often made without sufficient patient input.

**SECTION 3: CHALLENGES OF ACCESSING TIMELY DIAGNOSTICS**

**Rate the significance of the below challenges to accessing TIMELY DIAGNOSTICS for individuals with rare diseases in your country**

Q.1. Limited expertise significantly affects the ability to accurately diagnose rare diseases, as there is a shortage of specialized knowledge and trained professionals.

Q.2. Insufficient laboratory and genetic testing resources significantly limit diagnostic capabilities and contribute to delays in obtaining accurate diagnoses.

Q.3. Challenges with availability and access, particularly in remote or underserved areas, exacerbate delays and limit the ability to obtain necessary diagnostic services.

Q.4. Delays in diagnosis and processing times lead to extended periods before a definitive diagnosis is made, adversely affecting treatment outcomes.

Q.5. High costs associated with diagnostic tests, combined with insurance coverage and reimbursement issues, create substantial financial barriers for patients seeking essential diagnostics.

Q.6. Early screening for rare diseases is often limited due to the unavailability of free tests provided by pharmaceutical companies and the challenges related to genetic testing costs.

Q.7. The lack of education and empowerment of medical students hinders awareness among the general population, which delays timely access to diagnostics for patients with rare diseases.

Q.8. Are there any additional challenges related to accessing timely diagnostics for rare diseases that have not been covered in our discussion?

- Lack of national screening programs.
- Sometimes care providers are not aware of the access and services available for individuals with rare diseases, leading to longer delays before people recognize their illnesses.
- The presence of neonatal screening programs, such as for Sickle Cell Disease (SCD), is essential.

**When presenting the challenges of disease recognition and diagnosis to a decision-maker or payer, which challenge, in your opinion, should be addressed as the top priority?**

- **Q.1. (n=40%):** Limited expertise significantly affects the ability to accurately diagnose rare diseases, as there is a shortage of specialized knowledge and trained professionals.
- **Q.6. (n=20%):** Early screening for rare diseases is often limited due to the unavailability of free tests provided by pharmaceutical companies and the challenges related to genetic testing costs.
- **Q.3. (n=10%):** Challenges with availability and access, particularly in remote or underserved areas, exacerbate delays and limit the ability to obtain necessary diagnostic services.
- **Q.4. (n=10%):** Delays in diagnosis and processing times lead to extended periods before a definitive diagnosis is made, adversely affecting treatment outcomes.
- **Q.5. (n=10%):** High costs associated with diagnostic tests, combined with insurance coverage and reimbursement issues, create substantial financial barriers for patients seeking essential diagnostics.

**SECTION 4: CHALLENGES OF ACCESS TO ADEQUATE TREATMENT**

**Rate the significance of the below challenges to accessing ADEQUATE TREATMENT for individuals with rare diseases in your country**

Q.1. The availability of medications, particularly cutting-edge and high-cost therapies, significantly impacts the ability to manage rare diseases effectively in our country.

Q.2. The cost of these therapies and the challenges related to insurance coverage and reimbursement pose a substantial barrier to accessing adequate treatments.

Q.3. Access to rare disease therapy is limited, as treatments are less likely to receive marketing authorization than drugs for non-orphan diseases, and the combination of high costs with weak or often lacking efficacy data impedes access to reimbursement.

Q.4. Expertise in rare diseases is limited, with HCPs lacking specific training and education on rare diseases and their management, which can compromise the quality and effectiveness of patient care.

Q.5. The critical lack of collaboration between multidisciplinary teams hinders the provision of optimal pharmacological and non-pharmacological treatment options.

Q.4. The absence of national or regional treatment guidelines further complicates the management of rare diseases, leading to inconsistencies in care.

Q.6. Limited education and awareness about the appropriate use of medications exacerbate these challenges, impacting treatment outcomes.

Q.7. Are there any additional challenges related to accessing adequate treatment for rare diseases that have not been covered in our discussion? (e.g., psychological treatment)

- HCP awareness is limited.
- There is insufficient information given to patients with rare diseases regarding how to take their medication, which can lead to improper use that may either cause harm or reduce its effectiveness.
- Lack of collaboration with international experts who have more exposure to rare diseases.
- Lack of support and coverage for mental health care for patients, caregivers, and affected family members.
- Insufficient coverage for physical therapy and other therapies that complement the treatment plan.
- Lack of coverage for medical devices and assistive tools.
- No access to psychological care for patients with rare chronic diseases.
- There is a need for all supportive treatments to be available in one center.

**When presenting the challenges of disease recognition and diagnosis to a decision-maker or payer, which challenge, in your opinion, should be addressed as the top priority?**

- **Q.1. (n=40%):** The availability of medications, particularly cutting-edge and high-cost therapies, significantly impacts the ability to manage rare diseases effectively in our country.
- **Q.2. (n=20%):** The cost of these therapies and the challenges related to insurance coverage and reimbursement pose a substantial barrier to accessing adequate treatments.
- **Q.4. (n=10%):** The absence of national or regional treatment guidelines further complicates the management of rare diseases, leading to inconsistencies in care.
- **Q.6. (10%):** Limited education and awareness about the appropriate use of medications exacerbate these challenges, impacting treatment outcomes.
- **Q.5. (10%):** The critical lack of collaboration between multidisciplinary teams hinders the provision of optimal pharmacological and non-pharmacological treatment options.
