# Supplementary material for: Management of patients with rare diseases in the Middle East: challenges & opportunities – insights from the Rare Advocacy Council
Source: Orphanet J Rare Dis. 2026 Jul 31;21:260. doi: 10.1186/s13023-026-04469-1 (PMC13428422; doi:10.1186/s13023-026-04469-1)
Supplement: Supplementary file 2 — Supplementary Material 2 [file 13023_2026_4469_MOESM2_ESM.docx]

**SUPPLEMENTARY MATERIAL 2**

**SECTION 1: Challenges of disease recognition and diagnosis**

**Proposed Solutions to Address the Absence of Registries for Rare Diseases: Challenge n.1.1**

**1.** **Establish a National Registry Framework**

- Initiate a pilot program focused on creating a registry for one specific rare disease. This initiative can serve as a model for broader implementation.
- Utilize collaboration and partnerships among patient advocacy groups (PAGs), healthcare providers, and relevant societies to ensure comprehensive data collection and engagement.
- Develop a centralized database that integrates data from multiple healthcare providers and facilities, ensuring uniformity through a standardized data template.

**2.** **Engage Stakeholders and Governance**

- Assign responsibility to higher management within the Ministry of Health (MOH) to act as key stakeholders in initiating and sustaining registries.
- Explore partnerships with pharmaceutical companies and hospital systems to support the creation of registries, particularly in regions without existing frameworks.

**3.** **Address Resource and Workload Constraints**

- Motivate clinicians and healthcare providers to contribute data by offering:
  - Protected time for research and data entry.
  - Financial incentives or manpower support to alleviate the burden of heavy clinical workloads.
- Form a dedicated group of clinicians and statisticians to create and maintain databases and registries while advocating their importance to stakeholders.

**4.** **Involve Patients in Registry Development**

- Actively involve patients in the process by providing them with:
  - Access to their data within the registry.
  - The ability to enter Patient-Reported Outcomes (PROs), such as Quality of Life (QoL) data, to enhance the registry’s value.

**5.** **Leverage Global and Regional Best Practices**

- Adopt successful registry models, such as the French National Registry for Rare Diseases (<https://www.bndmr.fr/>), as a template for development.
- Learn from initiatives like the European Reference Networks (ERNs), which demonstrated the importance of proving value before expanding registries across disease fields (<https://eurreb.eu/>).

**6.** **Promote Sustainability and Multidisciplinary Collaboration**

- Ensure long-term registry viability by avoiding redundancy and fostering collaboration within the same disease field.
- Implement a multidisciplinary approach that involves healthcare professionals across different fields, including geneticists, statisticians, and clinicians, to ensure comprehensive patient care and robust registry development.

**7.** **Enhance Public Awareness and Legislative Support**

- Raise community awareness about rare diseases by utilizing widely accessible educational tools to enhance recognition and data reporting.
- Strengthen genetics legislation to standardize rare disease identification and promote stepwise approaches to patient care.

These solutions are illustrated in **Figure 3**.

**Proposed Solutions to Address the Insufficient Use of Media: Challenge n.1.2**

**1. Strategic Collaboration with Media Outlets**

- Partner with mainstream media (TV, radio, and newspapers) to produce educational campaigns focused on rare diseases tailored to different demographics.
- Target younger populations through short, visually appealing videos, using digital platforms like YouTube, TikTok, and Instagram.

**2. Development of Inclusive and Accessible Content**

- Create audiovisual materials with simplified language, visuals, and accessibility features (e.g., subtitles, audio descriptions, and sign language).
- Use storytelling techniques and real-life patient testimonials to make the content relatable and emotionally engaging.

**3. Leveraging Influencers and Community Leaders**

- Collaborate with influencers, celebrities, or trusted community leaders to amplify awareness campaigns, ensuring the content resonates with their audience.

**4. Age-Specific Education Campaigns**

- Design social media campaigns targeting younger audiences, incorporating interactive elements such as quizzes or challenges to effectively engage them.
- For older populations, prioritize traditional media, such as television or community radio programs, to ensure accessibility and trust.

**5. Centralized Content Creation and Dissemination**

- Establish a centralized team involving public health authorities, patient advocacy groups, and media experts to design consistent and unified awareness campaigns.

**6. Public Health Support and Funding**

- Advocate for funding from public health departments to create professional, high-quality audiovisual content.
- Promote partnerships with educational institutions to develop materials as part of student-led projects or community outreach programs.

**7. Measuring Impact and Engagement**

- Use analytics and audience feedback to evaluate the reach and effectiveness of campaigns, enabling continuous improvement.
- Conduct surveys or focus groups among target demographics to ensure content relevance and impact.

**8. Patient Involvement in Decision-Making and Media Campaigns**

- Involve patients more actively in decision-making processes related to media campaigns. Having patients share their personal experiences helps humanize the disease and ensures a more relatable message.
- **Campaigns by Pharmaceutical Companies**: Pharmaceutical companies should be encouraged to run media campaigns, particularly focusing on audiovisual materials that raise awareness about rare diseases. These campaigns should target both younger and older generations, addressing the knowledge gap across age groups.
  1. **Collaboration with Scientific Networks**
- Collaborate with organizations such as Endo-ERN, which works with societies like the European Society for Endocrinology (ESE) and the European Society for Paediatric Endocrinology (ESPE). This can lead to stronger partnerships with scientific associations that are knowledgeable in specific disease fields, allowing for more targeted educational efforts.
- **Global and Regional Networks**: Other regions should consider setting up similar collaborative networks to ensure the widespread distribution of media content that educates the public about rare diseases (**Figure 3**).

**SECTION 2: CHALLENGES OF LIVING WITH THE DISEASE & THE CONTINUUM OF CARE**

**Proposed Solutions to Address Limited Patient Representation: Challenge n.2.1**

- **Prioritize Quality of Life and Holistic Approaches**
  - Prioritize the quality of life for individuals with rare diseases, addressing needs beyond just medical management.
  - Incorporate a multidisciplinary approach with professionals like clinical social workers, clinical coordinators, and clinical psychologists, particularly those specialized in clinical genetics.
  - Continuously ask, "How can this patient thrive outside the hospital?" to identify and address their broader needs.
  - Strengthen collaboration among ministries (health, education, and social services) to better address the diverse needs of patients with rare diseases.
- **Legalize and Empower Patient-Led Groups**
  - Legalize and empower patient-led groups to ensure the patient's voices are properly represented in decision-making processes.
  - Encourage collaboration between doctors and patient organizations, ensuring that patient engagement is an integral part of healthcare discussions.
- **Increasing Awareness Among Healthcare Providers**
  - Improve care providers' understanding and increase doctors' awareness of the importance of patient partnership and engagement in care decisions.
- **Promote the Involvement of Educated Patients & Empower them as Ambassadors**
  - Select young patients to be representatives, as they are often more motivated and equipped to engage in advocacy and awareness campaigns.
  - Identify and empower some key patients with rare diseases to act as ambassadors in patient groups and advocacy campaigns.
  - Example: In 2012, a PhD patient with amyloidosis began leading a patient group and became an ambassador, raising awareness and educating others about treatment options and disease understanding.
- **Strengthen Patient Organization Infrastructure**
  - Acknowledge the lack of functioning patient organization infrastructure that hinders collaboration. Encourage doctors to assist active patients in founding patient organizations, which can be critical in helping with information dissemination and advocacy.
  - Example: In 1996, doctors helped active patients by providing them with information to create leaflets and website content and speaking at patient meetings.
- **Support for Training and Capacity Development**
  - Provide training sessions on capacity development for patient organizations to strengthen their capabilities to advocate and collaborate effectively with healthcare professionals.
  - Organize discussions between doctors and patient representatives to foster mutual understanding of the benefits of collaboration. Exchange ideas with international doctors who work closely with patient organizations.
- **Funding for Patient-Led Initiatives**
  - Address the issue of funding patient-led initiatives by seeking grants and organizational support from healthcare institutions and charities to help cover expenses for events, meetings, and awareness campaigns.
- **Address the Challenge of Active Patients' Responsibilities**
  - Acknowledge the challenges faced by active patients and their families, who may be balancing advocacy work with personal responsibilities such as caring for other children or managing their health.

**Figure 5** provides an overview of these solutions.

**Proposed Solutions to Address Lack of Collaboration Between Multidisciplinary Teams: Challenge n.2.2**

- **Create Centers of Excellence for Rare Diseases**
  - Establish regional or national centers of excellence for rare diseases that centralize expertise and resources, modeled after successful examples, such as pituitary centers of excellence where integrated multidisciplinary teams (MDTs) deliver coordinated, expert patient care.
- **Encourage the MDT Approach**
  - Implement a multidisciplinary approach for rare disease management that ensures comprehensive care, with MDTs established within specialized centers and academic hospitals, which offer a collaborative environment for various specialties.
- **Allocate Time for Collaboration**
  - Allocate protected time for clinicians to meet and collaborate, ensuring that MDT meetings are not compromised by competing clinical schedules.
  - Involve hospital administration in prioritizing team-based approaches by raising awareness of the added value MDTs bring to the management of rare disease patients.
- **Foster National and International Collaboration**
  - Collaborate with international organizations to access global expertise and promote best practices.
  - Support healthcare professionals in participating in international congresses and identifying international mentors who can strengthen their capacity to improve the patient journey.
  - Recognize and reward expertise by ensuring adequate compensation for professionals working within interdisciplinary teams.
  - Encourage involvement in international forums by inviting professionals to global congresses and facilitating mentorship opportunities to enhance the patient journey.
- **Training Coordinators to Optimize Collaboration**
  - Train coordinators to optimize collaboration within and between MDTs, improving communication and scheduling.
- **Promote System Change Towards Interdisciplinary Approaches**
  - Advocate for a system change to institutionalize the interdisciplinary approach for rare diseases, ensuring policies support MDT operations in centers of expertise.
- **Recognize Expertise and Offer Adequate Incentives**
  - Provide adequate compensation to professionals working in interdisciplinary teams, recognizing their expertise and commitment to collaborative care.
  - Highlight and promote successful models of MDT approaches, such as the pituitary centers of excellence, to demonstrate their impact on patient outcomes and care coordination.
- **Utilize the Pyramid of Patient Organization Development**
  - Integrate the adapted pyramid of Maslow to guide the development phases of patient organizations, offering them a framework for growth.
- **Develop Unified Infrastructure and Referral Pathways**
  - Develop well-defined referral pathways between public and private sectors to ensure timely access to specialized care and improve coordination among healthcare providers (**Figure 5**).

**SECTION 3: CHALLENGES OF ACCESSING TIMELY DIAGNOSTICS**

**Proposed Solutions to Address Limited Expertise for Diagnosis: Challenge n.3.1**

1. **Expand Specialist Education and Training:**
   - Develop residency, fellowship, and subspecialty programs focused on rare diseases.
   - Provide financial incentives, such as scholarships or grants, to encourage healthcare professionals to specialize in rare diseases.
   - Establish mentorship programs where experienced specialists in rare diseases guide young professionals.
2. **Utilize Telemedicine for Knowledge Sharing:**
   - Implement telemedicine platforms to connect local practitioners with experts in rare diseases for real-time consultations and case discussions.
   - Organize virtual conferences and webinars to share the latest advancements in diagnostics and management.
3. **Create Multidisciplinary Diagnostic Hubs:**
   - Develop regional centers of excellence with dedicated multidisciplinary teams, including geneticists, bioinformaticians, and biochemical specialists.
   - Equip these hubs with advanced diagnostic tools and provide them with sustainable funding to ensure continuity.
4. **Encourage International Collaboration:**
   - Establish partnerships with global centers of excellence to facilitate exchange programs, observerships, and access to cutting-edge expertise.
   - Invite international experts to conduct training sessions and workshops locally.
5. **Support Young Professionals & Recognize Expertise Development:**
   - Provide young healthcare professionals with opportunities to attend international congresses and connect with mentors.
   - Encourage early exposure research and clinical care for rare diseases to spark long-term interest and expertise development.
   - Provide recognition and career advancement opportunities for healthcare providers specializing in rare diseases.
   - Introduce awards or certifications for professionals contributing who contribute to the diagnosis and research of rare diseases.
6. **Build National and Regional Networks:**
   - Create a centralized directory of rare disease specialists and diagnostic facilities for streamlined referrals.
   - Facilitate regular discussions and case reviews among healthcare providers through online networks and collaborative platforms.
7. **Enhance Awareness Among Healthcare Professionals:**
   - Integrate rare disease topics into medical school curricula and continuing medical education (CME) programs.
   - Organize grand rounds or case presentations to showcase real-world cases of rare diseases and their diagnostic approaches.
8. **Promote Data Sharing and Research:**
   - Develop centralized databases to collect and share patient data, enabling pattern recognition and better diagnostic support.
   - Collaborate with research institutions to study rare disease patterns and identify biomarkers for improved diagnosis (**Figure 7**).

**Proposed Solutions to Address Limited Early Screening in Diagnosis: Challenge n.3.2**

1. **Strengthen Stakeholder Collaboration**:
   - Engage insurance companies, policymakers, and pharmaceutical companies to ensure affordable and accessible genetic testing.
   - Advocate for long-term partnerships with pharmaceutical companies to expand free or subsidized test programs.
2. **Implement Targeted Screening Programs**:
   - Focus on diseases with higher prevalence or actionable outcomes to maximize the impact of early screening efforts.
   - Organize pilot projects for selected diseases with pharmaceutical support to demonstrate feasibility and efficacy.
3. **Increase Awareness and Education**:
   - Educate healthcare professionals, including nurses and physicians, on the availability and processes of free genetic tests.
   - Develop workshops and materials to improve the efficiency of test administration in hospitals.
4. **Centralize Testing Efforts**:
   - Establish regional centers for rare disease screening to consolidate resources and expertise.
   - Integrate telemedicine platforms for test result analysis and expert consultations.
5. **Develop National Screening Programs**:
   - Advocate for the introduction of comprehensive newborn screening and premarital screening programs.
   - Create a national framework for genetic testing to ensure consistency and accessibility.
6. **Policy and Financial Support**:
   - Work with governments and insurance companies to develop policies covering genetic test costs.
   - Highlight the long-term cost benefits of early screening to gain support from payers and stakeholders. This objective can be supported by initiating a study on the social impact of rare diseases in the absence of such programs, underscoring the need for actionable solutions.
7. **Leverage International Models**:
   - Study successful screening programs in Europe and other regions to replicate best practices.
   - Foster international collaboration for knowledge exchange and capacity building.
8. **Promote Public Awareness Campaigns**:
   - Conduct awareness initiatives to address cultural and social barriers to genetic testing, such as those related to consanguinity.
   - Emphasize the importance of early screening in reducing the burden of rare diseases.
9. **Enhance Data Sharing and Interpretation**:
   - Develop centralized databases for genetic testing results to improve pattern recognition and rare disease diagnostics.
   - Train specialists in interpreting genetic data to bridge expertise gaps.
10. **Support Research and Innovation**:
    - Encourage research on cost-effective and targeted genetic testing approaches.
    - Partner with pharmaceutical companies to fund the development of innovative diagnostic tools (**Figure 7**).

**SECTION 4: CHALLENGES OF ACCESS TO ADEQUATE TREATMENT**

**Proposed Solutions to Availability of Medications: Challenge n.4.1**

**1.** **Policies and Regulations**

- Streamline and expedite approval processes for orphan drugs to avoid delays in their market availability.
- Encourage governments to collaborate with academic and private institutions to share the costs and risks of developing expensive therapies.

**2.** **Collaboration and Partnerships**

- Support clinical trials and research collaborations in strategic regions, such as the United Arab Emirates, to introduce innovative therapies.
- Foster international collaborations to share resources and financial burdens associated with managing rare diseases.

**3.** **Healthcare System Strengthening**

- Encourage pharmaceutical companies to balance the availability of innovative treatments with affordable generic medications for comprehensive patient care.
- Ensure that a focus on cutting-edge treatments does not overshadow access to basic, affordable therapies. Patients with rare diseases often suffer from multiple conditions, making the availability of all essential medicines crucial. Limited access to low-cost medications can exacerbate the challenges of managing rare diseases effectively.
- Companies offering high-cost, innovative therapies should also be encouraged to take responsibility for maintaining the supply of affordable generic medicines, ensuring comprehensive patient care across all socioeconomic groups.
- Inform future decisions by conducting impact studies comparing patient quality of life with and without access to rare disease treatments.

**4.** **Awareness and Capacity Building**

- Increase awareness among healthcare providers about rare disease treatments and their importance in enhancing early diagnosis and management.
- Organize training programs and promote knowledge sharing to ensure effective use of available resources.

**Proposed Solutions to Cost and Reimbursement of Medications: Challenge n.4.2**

**1. Policies and Regulations**

- Advocate for policy adaptations requiring acceptable coverage of life-saving therapies by health insurance companies.
- Expand formulary approvals to include essential therapies for rare diseases.
- Encourage bulk purchasing agreements between governments and manufacturers to reduce costs.
- Reevaluate pricing structures associated with treatments for rare diseases, particularly for populations in developing countries who often cannot afford the treatment costs prevalent in their regions.
- Lobby policymakers and insurance companies for improved coverage policies, ensuring access to necessary treatments for rare diseases.
- Introduce government-backed initiatives to facilitate affordable access to treatments for rare diseases, including international collaborations to reduce costs.

**2. Collaboration and Negotiations**

- Facilitate direct negotiations between payers (government or private) and manufacturers to achieve fair pricing.
- Promote regional collaboration in Health Technology Assessments (HTA) to ensure consistent decisions across neighboring countries.
- Foster collaborative partnerships between pharmaceutical companies and charitable organizations to facilitate access to essential treatments for individuals in need.
- Negotiate fair pricing agreements with pharmaceutical companies, ensuring that pricing reflects regional economic realities and patient needs.

**3. Patient Advocacy and Inclusion**

- Leverage the patient voice as a negotiation tool with insurance companies and policymakers to emphasize the importance of access to therapies.
- Advocate for mandatory patient involvement in HTA decisions and regulatory processes to ensure patient needs and quality of life enhancements are prioritized.
- Support programs that provide funding for patients who cannot afford their medications and offer opportunities for others to receive free medications.

**4. Funding Strategies**

- Develop pricing models that link drug costs to their corresponding health outcomes, enabling patients to pay based on the effectiveness of their treatment.
- Encourage pharmaceutical companies to lower prices for low-income countries or offer sponsorship programs.
- Implement funding strategies that support equal access to both high-cost and essential therapies for patients with rare diseases.

**5. Education and Awareness**

- Educate the public about the appeal process for insurance claims related to rare disease treatments.
- Enhance awareness among decision-makers about the unmet needs of patients and the value of new therapies in improving quality of life.
- Promote awareness of programs and initiatives that enable patients to access necessary treatments, including awareness about insurance claims processes and patient assistance programs.

**6. Government and Organizational Support**

- Advocate for government-sponsored coverage in countries where the cost of therapies is prohibitive.
- Use concerned medical committees to lobby for better insurance regulations and improved reimbursement frameworks.
- Foster international collaboration to reduce costs and share resources, ensuring that patients in all regions can access necessary therapies.

Solutions for the main two challenges are illustrated in **Figure 9**.
